# Data-Prefetching Scheme Based on Playback Delay and Positioning Satisfaction in Peer-To-Peer Video-On-Demand System

**DOI:** 10.3390/s18030816

**Published:** 2018-03-08

**Authors:** Lei Wang, Xiaorui Li, Yaqiu Liu, Guan Gui

**Affiliations:** 1 National and Local Joint Engineering Laboratory of RF Integration and Micro-Assembly Technology, Nanjing University of Posts and Telecommunications, Nanjing 210003, China; wanglei@njupt.edu.cn (L.W.); 1016010627@njupt.edu.cn (X.L.); liuyq@shrc.gov.cn (Y.L.); 2 National Mobile Communications Research Laboratory, Southeast University, Nanjing 210096, China; 3 Shanghai Radio Monitoring Station, Shanghai 200031, China

**Keywords:** video-on-demand (VoD), video cassette recorder (VCR), multiple-attribute decision-making (MADM), playback delay, positioning satisfaction

## Abstract

As one of the most important applications in peer-to-peer (P2P) networks, the video-on-demand (VoD) system freely supports video cassette recorder (VCR) operation for users. However, the users may experience significant playback delay after frequent VCR operations in the VoD system, which will affect the quality of experience (QoE) of the users. Hence, selecting an appropriate data-prefetching strategy to support better VCR operation is an important approach to improve the QoE. This paper proposes a data-prefetching strategy (DSA) to determine the most suitable anchor interval by considering the playback delay and positioning satisfaction. According to the DSA, we use the multiple-attribute decision-making (MADM) theory to model the selection of intervals of prefetching data blocks (i.e., anchor interval) and the technique for ordering preference by similarity to an ideal solution (TOPSIS) algorithm to solve the MADM. The simulation results show that the DSA strategy obtains higher positioning satisfaction than the existing schemes, which is approximately 60% higher than the anchor points, popular parts of video, and user interests (API)-based method. Moreover, with the increase in network bandwidth, the DSA strategy can minimize the playback delay after VCR operation using relative few extra bandwidths.

## 1. Introduction

In recent years, with the rapid development of internet and multimedia technology, increasingly more users have gained access to high-speed internet, and the service of online video-on-demand (VoD) [1] has been rapidly developed. Currently, one of the most common methods to support video-streaming services is peer-to-peer (P2P) networks. In P2P-based VoD systems [2], each peer can download and watch videos from the server or other peers, and provide the downloaded video segments to other requesting peers, which can improve the system’s service capabilities and reliability and reduce the server load and network construction costs.

Compared with the live video system, a significant feature of the VoD system is that it can support the users to perform video cassette recorder (VCR) operations [3] (e.g., randomly dragging the progress bar for seeking, pause, fast forward, and rewind). In the P2P-based VoD system without data-prefetching technology, the users may experience significant playback delay after frequent video cassette recorder (VCR) operations, which seriously affects the users’ viewing experience. Hence, the most serious challenge in the design of P2P-based VoD systems is to find a suitable segment selection algorithm to reduce the playback delay after the VCR operation. A data-prefetching strategy was proposed, i.e., if residual download bandwidth remains, the peer can prefetch a part of non-urgent data after the urgent data has been downloaded. The urgent data can ensure the video is continuously played in the next several minutes, and the non-urgent data is used to reduce the playback delay after the users perform VCR operations. In this strategy, the peer uses the residual bandwidth to download the data ahead of the current playback point, which can reduce the playback latency and effectively improve the viewing experience of users [4].

In [5], Yu et al. analyzed the VCR operation behaviors of users in large-scale VoD systems, and proposed a strategy to reduce the playback delay after the VCR operation by prefetching videos with high play counts. This strategy assumes that all users in the system have identical preferences, so they attempt to predict the video that is most likely to be played in the next few minutes by analyzing a large volume of user viewing logs. However, the assumption has drawbacks. For example, the viewing preferences of users vary for different times. Hence, inaccurate predictions will cause a low bit rate of prefetched data and waste bandwidth resources. In [6], the authors proposed an anchor-prefetching strategy. The anchor is distributed throughout the video file with a fixed interval (5 min), and each anchor consists of 10 continuous seconds. This traditional anchor-prefetching strategy can effectively reduce the playback delay after the VCR operation and avoid the problem of bandwidth waste caused by inaccurate prediction. The authors of [7] developed a prefetching strategy of API by prefetching both anchor points and popular segments of the video. This approach is complementary to the anchor-prefetching strategy, which can reduce the playback delay and improve the user satisfaction. However, to implement this strategy, it is necessary to detect the video segments that are currently played by each online P2P node, which will consume many extra bandwidth resources.

In [8], the authors measured and analyzed the rules of VCR operation when the user watches the video. The forward- and backward-jump lengths have been modeled with a Weibull distribution. The forward and backward distances have different parameter values of Weibull distribution. In addition, for different lengths of video files, the Weibull distribution parameter values also vary. This is the theoretical basis of our study on the data-prefetching strategy.

A new strategy to determine the most reasonable anchor interval is proposed in this paper by considering two factors of playback delay and positioning satisfaction. How to minimize the playback delay after the VCR operation is one of the most serious challenges in the design of P2P-based VoD systems, as mentioned above. And as mentioned in [6], in a VoD system with the anchor-prefetching strategy, the playback point is adjusted to the beginning of the closest anchor after the VCR operation. The disparity between the actual playback point and the designated point by the VCR operation may reduce the user’s viewing experience. Therefore, we introduce the concept of positioning satisfaction to reflect the user’s appraisal of the anchor-based VoD system caused by the disparity. Then, the user’s viewing experience is studied by considering the factors of playback delay and positioning satisfaction. The playback delay is a cost factor, and a lower value indicates better viewing experience of the users. The positioning satisfaction is a benefit factor, and a higher value indicates better viewing experience of the users.

The problem of selecting the most reasonable anchor interval was modeled using the multiple-attribute decision-making (MADM) theory [9]. Based on the factors of playback delay and positioning satisfaction, the MADM model is established. By solving this model using the technique for ordering preference by similarity to an ideal solution (TOPSIS) method, the optimal anchor interval is selected from 20 optional anchor interval schemes.

The remainder of the paper is structured as follows. The model of the P2P-based VoD system is described in Section 2. The problems studied in this paper are described in Section 3. The proposed optimal selection strategy of anchor interval is presented in Section 4. The performance evaluation is presented in Section 5. Section 6 concludes this paper.

## 2. System Model

Before stating the research of the data-prefetching strategy, it is necessary to introduce the P2P-based VoD system. This system is an overlay network [10] built between the terminals and the integration content distribution network (CDN) [11]. It can automatically distribute the content and segment the traffic flow. This system can transmit the videos in CDN to P2P terminals (i.e., client node, CN) and share videos on the CN using specified strategies.

**Figure 1:**
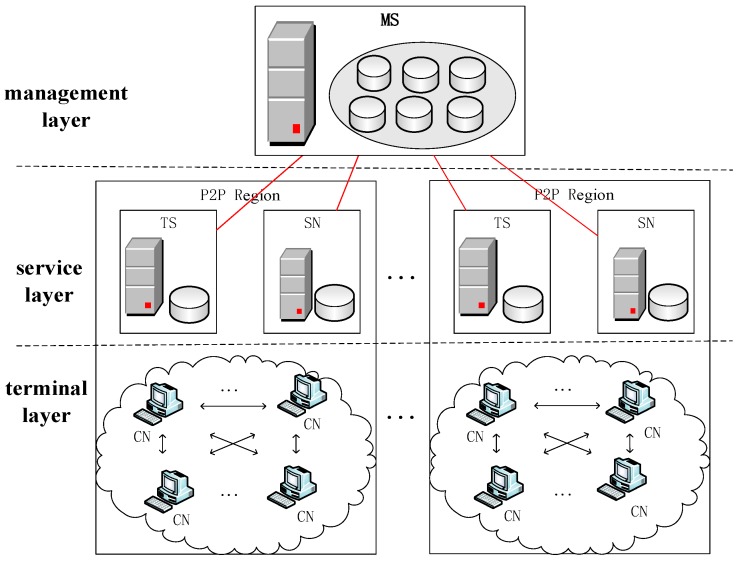
Architecture of the peer-to-peer (P2P)-based video-on-demand (VoD) system.

As illustrated in Figure 1, this communication system [12] is divided into three layers: management layer, service layer, and terminal layer. The management layer is deployed on the backbone network, and mainly includes a P2P management subsystem (MS), which is responsible for managing all P2P regions. It collects the operation data of the network element in the service layer and displays the system status through web pages, which includes the peer online status, video distribution information, etc. In addition, it can provide the query function; each provincial or municipal network is divided into a P2P region. In each P2P region, a P2P super node (SN) and a P2P tracker subsystem (TS) are deployed to form the service layer. The SN is responsible for importing videos into the P2P system, splitting and managing the videos in the system, and providing video segments for other peers. The TS is mainly responsible for the management of CNs in a P2P region. Meanwhile, the TS continues the video directory management to support video positioning. The terminal layer consists of numerous CNs, which can be mobile phones [13], tablets, desktops, etc. The CN can download and play videos and provide video segments for other CNs in the same P2P region. However, this system cannot support inter-region data exchange among the CNs.

When a CN goes online, it must first register with the TS. After the registration is successful, the servable video list of this region is sent to this CN by the TS. The users can select the video that interests them from the video list. After receiving the user’s selection, the CN must determine the download order of the video segments according to the segment selection algorithm. Then, the CN requests the servable peers from the TS and sorts the servable nodes. After selecting the most suitable node as the content provider, the CN can download video segments in order. It is notably important to improve the user’s viewing experience by determining a suitable order to download the video segments, which we will introduce in detail in the following chapters.

## 3. Detailed Problem Formulation

The segment-downloading sequence affects the viewing experience of the user in the P2P-based VoD system. Therefore, many segment selection strategies were proposed. To support the particularity of the VCR operation in the P2P-based VoD system, data-prefetching strategies were introduced as a specific segment selection strategy, i.e., downloading the data before the current playing point in advance, which is designed to reduce the playback delay after VCR operations. The most mature data-prefetching strategy is the anchor point strategy [6]. When the user watches a video, the CN with the anchor-prefetching strategy downloads the anchor data with the residual download bandwidth after downloading the urgent data. When the user jumps to a new point of the video using a VCR operation, the playback point is adjusted to the beginning of the closest anchor. If the anchor has been downloaded, the CN will resume playing the video with no delay. In this paper, we hope to improve the viewing experience of the user after a VCR operation by improving the anchor-prefetching strategy. To improve this strategy, we consider two aspects: anchor duration and anchor interval.

First, we discuss the anchor duration. Each anchor must be a block that can be independently played. Based on the content fragmentation strategy in the P2P-based VoD system in Section 2, a chunk size is approximately 2 M [14]. An Android native player must download approximately 2 chunks before it begins playing a video, i.e., the data block has at least 4 M bits that can be independently played. According to the research of PPLive, the video code rate is 381–700 kbps. Therefore, to satisfy the requirement of the player to play the video segments, each anchor duration is 6–10 s. To ensure the normal playback of video with different bit rates, we set the anchor duration to 10 s.

Second, to study the anchor interval, we must consider two factors: playback delay and positioning satisfaction.

### 3.1. Playback Delay

**Figure 2:**
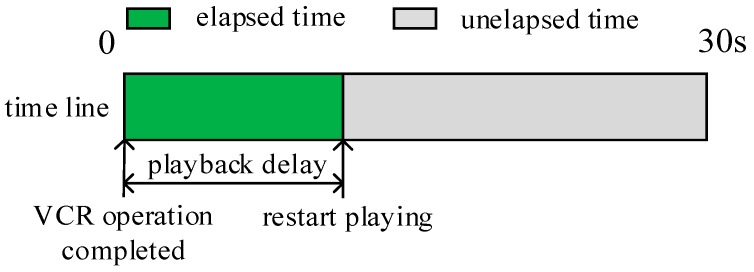
Playback delay.

The playback delay is the time the CN takes to resume playing the video after a VCR operation, as illustrated in Figure 2. A smaller playback delay indicates a better viewing experience.

When the anchor duration is fixed, if the anchor interval increases, the anchors that are perfected with identical residual download bandwidths will have a wider distribution in the video. When the user randomly jumps to a certain point using a VCR operation, the probability that the anchor near the point that has been downloaded increases, and the probability that the user experiences a delay decreases. Therefore, the average playback delay decreases.

We simulated the VCR behavior of a VoD system. The fast-forward and -backward operations compose a notably small percentage (approximately 1%) of the user’s actual operations. As mentioned in [15,16], most of the user interactions are forward search (48%) and pause (51%). Most VCR operations can be achieved through the jump process: jump to a new point from the current play point and resume playing the video. One search or pause must only jump once, whereas fast forwards or backwards usually consist of a series of jump processes [17]. Therefore, to study the playback delay of the VCR operation, this paper mainly pays attention to the forward-search jump behavior when the users are watching long videos.

The length of each jump in the VCR operation complies with Weibull distribution [8], and the probability density function is as follows:

(1) $$f(x,\alpha ,\beta )=\frac{\alpha }{\beta }{\left(\frac{x}{\beta }\right)}^{\alpha -1}e^{-{\left(\frac{x}{\beta }\right)}^{\alpha }}$$

Table 1 shows the parameters of the distribution.

**Figure 3:**
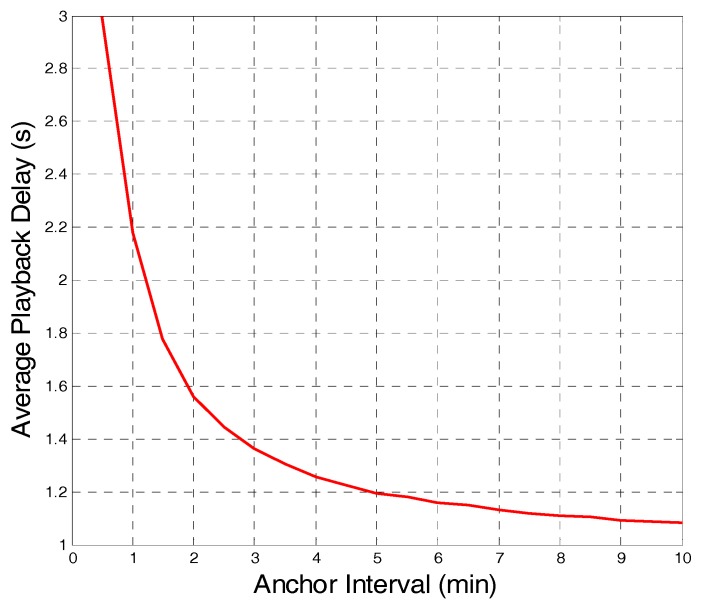
Average playback delay with different anchor intervals.

After the video was played for 1 min, the user randomly performed 20 VCR operations; then, the average playback delay was recorded. As shown in Figure 3, with the increase in anchor interval, the playback delay gradually decreases. When the users watch a video on demand, the delay should be as short as possible. From this perspective, a larger anchor interval corresponds to a better user experience.

### 3.2. Positioning Satisfaction

When the user performs a VCR operation, he/she hopes that the player resumes playing the video exactly from the designated point. As mentioned in the anchor-prefetching strategy, the actual playback point of the player does not exactly coincide with the designated point by the user. Thus, the concept of positioning satisfaction is generated.

**Figure 4:**
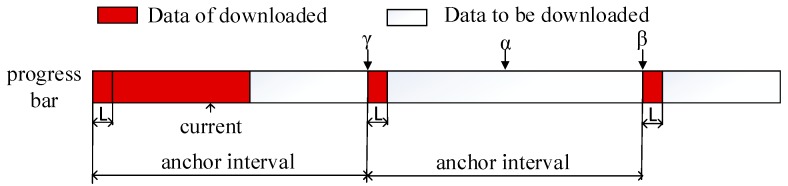
Seeking in a video.

As shown in Figure 4, α is the middle of the anchor interval. Therefore, if the user jumps to point α after a VCR operation, the worst case occurs, and the positioning satisfaction is minimal. However, if the user jumps to point γ or β, because the anchors have been perfected, the best case occurs, and the positioning satisfaction is 1 (i.e., the maximum value). By closing from α to β (or γ), the positioning satisfaction is increased.

Satisfaction is a fuzzy concept of people psychology, so we use the membership function of fuzzy mathematics to define the positioning satisfaction. We define that the positioning satisfaction is 1 if the time gap of the actual playback point and the designated point is less than 5 s, and a greater difference implies lower positioning satisfaction. The positioning satisfaction with time gap is calculated according to Equation (2):

(2) $$S(x)=\{\begin{array}{cc} 1, & x\leq 5s\\ e^{-{\left(\frac{x-5}{\lambda }\right)}^{2}}, & x>5s \end{array}$$

where λ is a constant strength factor.

**Figure 5:**
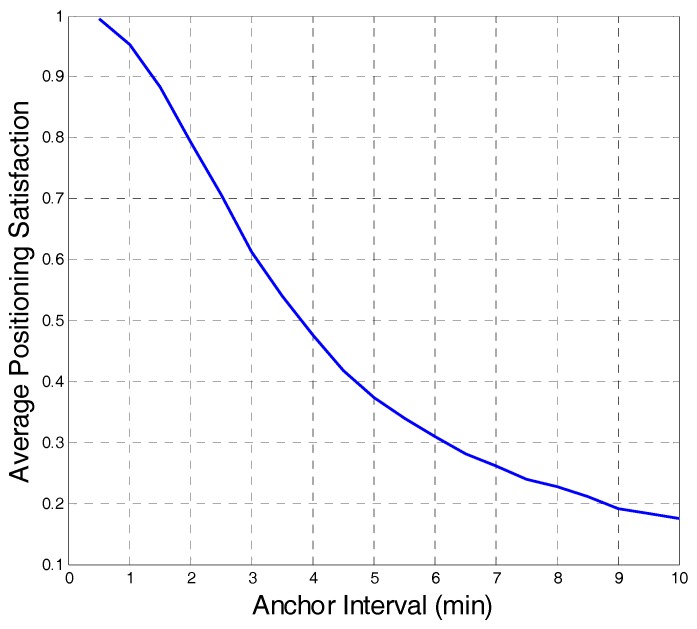
Average satisfaction with different anchor intervals.

We have studied how the anchor interval affects the positioning satisfaction using simulations. Since the user more likely jumps forward for a VCR operation [18,19]. We mainly focus on the forward-search behavior. After the video was played for 1 min, the user randomly performed 20 VCR operations; then, the average positioning satisfaction was recorded. As shown in Figure 5, with the increase in anchor interval, the positioning satisfaction gradually decreases. From this perspective, a smaller anchor interval corresponds to higher positioning satisfaction and better system performance.

It is not acceptable for the user to experience a long playback delay when they perform a VCR operation, i.e., a shorter playback delay corresponds to a better user experience. According to the analysis in Section 3.1, a larger anchor interval is better.

The positioning satisfaction reflects the difference between the actual playback point and the designated point. A smaller difference indicates higher satisfaction of the user. According to the analysis in Section 3.2, a smaller anchor interval is better.

Therefore, the playback delay and positioning satisfaction are two conflicting factors.

## 4. Anchor Optimization Selection Strategy

As analyzed, the playback delay is a cost factor, and the positioning satisfaction is a benefit factor. Based on the two types of factors, we present the anchor optimization selection strategy DSA. This paper aims at selecting the most suitable scheme from the limited number of optional anchor interval schemes. Hence, it is a typical MADM problem.

As one of the most widely used method to solve the MADM problem, the TOPSIS method [20] proposes the concept of ideal solution (*S*^+^) and negative ideal solution (*S*^−^). The basic principle of TOPSIS is using the distance from each candidate to *S*^+^ and *S*^−^ as the criterion to rank the candidates. According to the ranking, we can select the most suitable candidate. The detailed steps of the TOPSIS method are summarized as follows:

**Step 1. Construct the normalized decision matrix A**

First, it is important to construct the original decision matrix before the normalization, and the decision matrix V with m candidates and n factors is shown in Equation (3):

In this paper, the decision matrix V consists of 20 optional schemes and 2 factors.

(3) $$V=\left[\begin{array}{cccc} x_{11} & x_{12} & \cdots & x_{1n}\\ x_{21} & x_{22} & \cdots & x_{2n}\\ \vdots & \vdots & \vdots & \vdots \\ x_{i1} & \cdots & x_{ij} & \cdots \\ \vdots & \vdots & \vdots & \vdots \\ x_{m1} & x_{m2} & \cdots & x_{mn} \end{array}\right]$$

where $x_{ij}$ is the *j*-th factor value of the *i*-th scheme. To eliminate the difference of these two factors, the decision matrix must be normalized. For the benefit factor and cost factor, the normalization method is shown in Equations (4) and (5), respectively.

(4) $$a_{ij}=\frac{x_{ij}-\min(x_{ij})}{\max(x_{ij})-\min(x_{ij})}$$

(5) $$a_{ij}=\frac{\max(x_{ij})-x_{ij}}{\max(x_{ij})-\min(x_{ij})}$$

Then, the normalized decision matrix A is obtained:

(6) $$A=\left[\begin{array}{cccc} a_{11} & a_{12} & \cdots & a_{1n}\\ a_{21} & a_{22} & \cdots & a_{2n}\\ \vdots & \vdots & \vdots & \vdots \\ a_{i1} & \cdots & a_{ij} & \cdots \\ \vdots & \vdots & \vdots & \vdots \\ a_{m1} & a_{m2} & \cdots & a_{mn} \end{array}\right]$$

**Step 2. Calculate the weight of each factor**

The entropy weight is calculated as follows:

(7) $$w_{j}=\frac{1-e_{j}}{n-\sum_{j=1}^{n}e_{j}}$$

In Equation (7), $e_{j}$ is the entropy of factor *j*:

(8) $$e_{j}=-\frac{1}{\ln n}(\sum_{i=1}^{n}f_{ij}\ln f_{ij})\;(0\leq e_{j}\leq 1)$$

In Equation (8), $f_{ij}$ is the characteristic proportion of the factor:

(9) $$f_{ij}=\frac{a_{ij}}{\sum_{i=1}^{m}a_{ij}}$$

**Step 3. Construct the evaluation matrix based on the entropy weight:**

(10) $$Z=\left[\begin{array}{cccc} a_{11} & a_{12} & \cdots & a_{1n}\\ a_{21} & a_{22} & \cdots & a_{2n}\\ \vdots & \vdots & \vdots & \vdots \\ a_{i1} & \cdots & a_{ij} & \cdots \\ \vdots & \vdots & \vdots & \vdots \\ a_{m1} & a_{m2} & \cdots & a_{mn} \end{array}\right]\left[\begin{array}{cccc} w_{1} & 0 & \cdots & 0\\ 0 & w_{2} & \cdots & 0\\ \vdots & \vdots & \vdots & \vdots \\ 0 & \cdots & w_{j} & \cdots \\ \vdots & \vdots & \vdots & \vdots \\ 0 & 0 & \cdots & w_{n} \end{array}\right]=\left[\begin{array}{cccc} z_{11} & z_{12} & \cdots & z_{1n}\\ z_{21} & z_{22} & \cdots & z_{2n}\\ \vdots & \vdots & \vdots & \vdots \\ z_{i1} & \cdots & z_{ij} & \cdots \\ \vdots & \vdots & \vdots & \vdots \\ z_{m1} & z_{m2} & \cdots & z_{mn} \end{array}\right]$$

**Step 4. Determine the ideal and negative ideal schemes**

This step determines the ideal scheme *S*^+^ and negative ideal scheme $S^{-}$.

(11) $$S^{+}=[z_{1}^{+},z_{2}^{+}\cdots ,z_{n}^{+}]=\{(\underset{i}{\max}\;z_{ij}|j\in J),(\underset{i}{\min}\;z_{ij}|j\in J^{\prime })|\begin{array}{c} i=1,\ldots ,m\\ j=1,\ldots ,n \end{array}\}$$

(12) $$S^{-}=[z_{1}^{-},z_{2}^{-}\cdots ,z_{n}^{-}]=\{(\underset{i}{\min}\;z_{ij}|j\in J),(\underset{i}{\max}\;z_{ij}|j\in J^{\prime })|\begin{array}{c} i=1,\ldots ,m\\ j=1,\ldots ,n \end{array}\}$$

where J is the set of subscripts of the benefit factor; $J^{\prime }$ is the set of subscripts of the cost factor.

**Step 5. Calculate the distance of each scheme from the ideal and negative ideal schemes**

(13) $$d_{i}^{+}=\sqrt{\sum_{j=1}^{n}{\left(z_{ij}-s_{j}^{+}\right)}^{2}}\;(i=1,\ldots ,m)$$

(14) $$d_{i}^{-}=\sqrt{\sum_{j=1}^{n}{\left(z_{ij}-s_{j}^{-}\right)}^{2}}\;(i=1,\ldots ,m)$$

**Step 6. Calculate the distance of each scheme from the ideal and negative ideal schemes**

(15) $$r_{i}=\frac{d_{i}^{-}}{\left(d_{i}^{-}+d_{i}^{+}\right)}$$

**Step 7. Sort the candidate schemes according to** $r_{i}$ **.**

In this study, we propose 20 optional anchor interval schemes, and the length of the interval is 0.5–10 min with uniform increments. The TOPSIS method is used to comprehensively evaluate the 20 schemes, and the priority order of the candidate schemes is shown in Table 2.

In the TOPSIS method, each factor value of the ideal solution is the optimal value of all candidates. In other words, the ideal solution has the smallest playback delay and highest positioning satisfaction among all candidate schemes. Therefore, the ideal solution can achieve the best viewing experience. Each factor value of the negative ideal solution is the worst value of all candidates. The negative ideal solution will ideally result in the worst viewing experience. In fact, the ideal solution and negative ideal solution do not exist in the proposed candidates. The best candidate has the farthest distance from the negative ideal solution and the shortest distance from the ideal solution. Therefore, the best candidate has the highest viewing experience among all candidates.

Table 2 shows that scheme 5 is optimal, and the anchor interval is 2.5 min. We name this data-prefetching strategy the DSA strategy considering the playback delay and positioning satisfaction.

## 5. Experiment Evaluation

To verify the rationality of the proposed strategy in this paper, we compared it with the most mature anchor strategy, whose anchor interval is 5 min [6], and the API strategy proposed in [7]. The DSA strategy proposed in this paper is an improvement strategy for the anchor strategy. The proposed scheme improved the anchor interval to 2.5 min by the anchor optimization selection strategy formulated in Section 4. These two strategies share the same data prefetching mode. Peers with these two strategies download the anchor data before the current playing point with the surplus download bandwidth after downloading the urgent data. The API strategy is also an improvement strategy for anchor strategy, which aims to increase the user satisfaction and reduce the playback delay. In this strategy, peers downloads both the anchor at intervals of 5 min and the video segments that are popular with users in the current system before the current playing point.

We implemented the P2P-based VoD system and data-prefetching algorithm using Java. In the comparative experiment, we simulated the VCR operation process when the user watches a video in a P2P-based VoD system, and the test video bit rate is 600 kbps. After the video was played for 1 min, the user randomly performed 5 times VCR operations every 15 s.

### 5.1. Playback Delay

In a P2P-based VoD system, the data-prefetching strategy is proposed to minimize the latency that the user must wait after the VCR operation. Therefore, the playback delay is one of the most important indicators to evaluate the validity of a data-prefetching strategy. For identical network bandwidths, a smaller delay indicates a better data-prefetching strategy.

**Figure 6:**
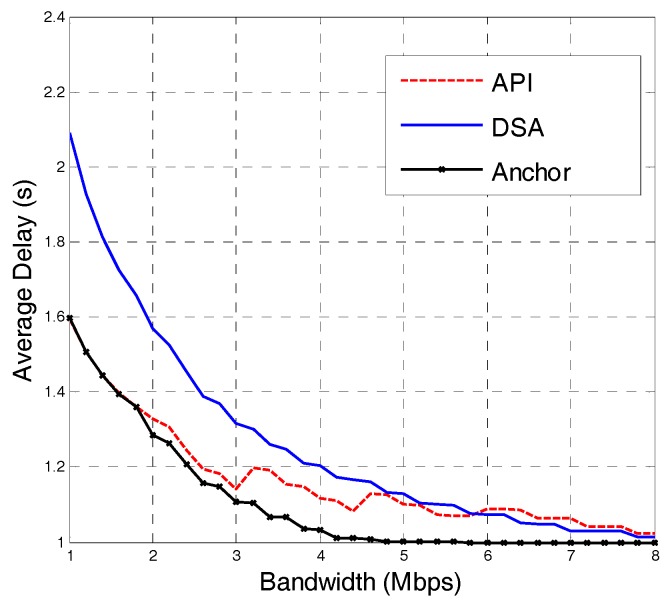
Average playback delay of different strategies.

As shown in Figure 6, when the network bandwidth is small, the DSA strategy requires the highest average delay, but it is only 0.5 s more than the other two strategies. With the increase in network bandwidth, the DSA strategy has the fastest performance improvement. When the network bandwidth increases to approximately 6 M, the average delay of the DSA strategy is lower than that of the API strategy, and almost equal to the anchor-prefetching strategy, which has the minimal value.

### 5.2. Positioning Satisfaction

The VoD system enables the users to perform VCR operations and quickly search for interesting content. The users hope that the player resumes playing the video exactly from their designated point after the VCR operation. Positioning satisfaction is the indicator to evaluate the proximity of the designated point and the actual playback point.

**Figure 7:**
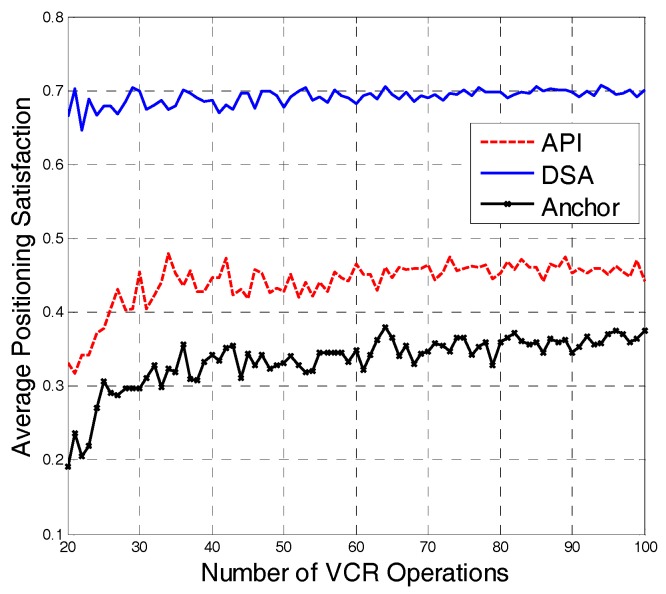
Average positioning satisfaction of different strategies.

As shown in Figure 7, with the increase in number of VCR operations, the positioning satisfaction of the three strategies basically remains stable. The API strategy prefetches the original anchor points and popular segments. To a certain degree, the API strategy improves the positioning satisfaction. However, compared with the API strategy, the proposed DSA strategy in this paper improves the positioning satisfaction by approximately 60%.

### 5.3. Extra Bandwidth Consumption

The peers in the P2P-based VoD system require auxiliary information to prefetch data, and the bandwidth consumed by the auxiliary information is the extra bandwidth. To prefetch data for the peers using the anchor-prefetching strategy or the DSA strategy, two extra fields must be added to the index file: the anchor length and the list of starting segment numbers for each anchor. When a peer begins watching an on-demand video, the index file from the server or peers must first be obtained and locally saved. Then, the peer requests the segments according to the index file and the user’s playing request. Therefore, to implement these prefetching strategies, one peer must only transmit two additional fields.

However, the API strategy prefetches both anchors and popular segments. To achieve this prefetching strategy, adding the two fields of the anchor length and starting segment number for each anchor to the index file is not sufficient, as previously mentioned. To acquire the popular segments of the current system, the server must perceive which segment of the video is currently played by each online peer. To satisfy this requirement, two information fields (the video ID and currently played video segment) should be added to the heartbeat packet, which is periodically sent by peers. The heartbeat packet is a collection of real-time basic information of the peers that must be periodically uploaded to the tracker server (TS) by online peers. The server manages the peers through the heartbeat packet information. A peer sends a heartbeat packet every 30 s by default.

**Figure 8:**
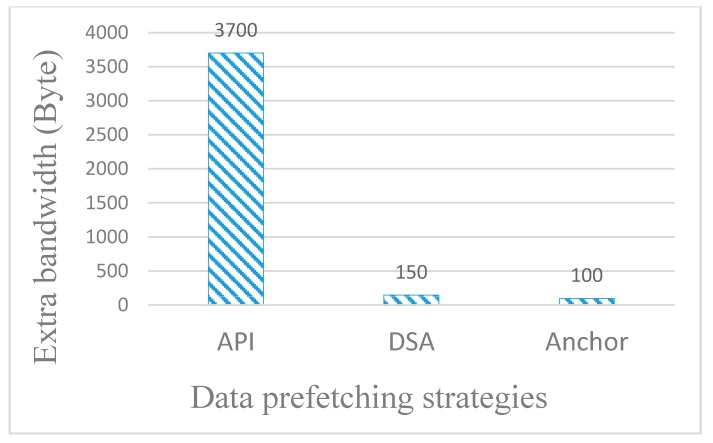
Extra bandwidth consumption of different strategies.

When an online peer watches a 60 min video, we assume that the user watches the video for a total of 30 min over a series of VCR operations. The extra bandwidth required for the three strategies is shown in Figure 8.

In the API strategy, the peer’s playing information must be added to the periodically sent heartbeat packet, which consumes 20–30 times more bandwidth than the other two strategies. However, the anchor-prefetching strategy and proposed DSA strategy in this paper consume notably little extra bandwidth for each video, which is within the acceptable range.

When tens of thousands of online peers watch thousands of on-demand videos in the P2P-based VoD system, the extra bandwidth consumed by the API sharply increases compared to the other two strategies. In other words, the implementation of the API causes a significant system burden. Therefore, the API has poor practicality.

## 6. Conclusions

In this paper, the optimal data-prefetching scheme is derived using the TOPSIS algorithm based on the MADM theory: the DSA strategy. In order to determine the most suitable data prefetching strategy, we considered factors of different type which can influence user’s viewing experience, such as playback delay and positioning satisfaction. The experiment demonstrates that the proposed DSA strategy in this paper improves the positioning satisfaction by approximately 60% relative to the API strategy. Moreover, the DSA strategy can ensure that the delay performance is relatively good. In addition, the delay performance increases the fastest with the increase in network bandwidth, compared with the other two strategies, and achieving this strategy requires less extra bandwidth. Therefore, the proposed DSA strategy in this paper has the best performance based on the comprehensive evaluation in these three strategies, and it can enable users to obtain a better viewing experience. Also, this paper has presented a model to determine the most suitable prefetching data strategy based on different factors. The model has good extensibility, and can be extended from two factors to multiple factors. This model can also be applied to the segment selection algorithm to determine the downloading order of different types of segments.

Due to the limited conditions, the experiments were evaluated by simulations with Java. Hence, some future work is still needed. The performance of the DSA strategy needs to be validated in real experimental environments.

## Figures and Tables

**Table 1:** Jump length characterization.

Interaction	Video Type	Distribution	Parameters
Forward jump length	Short videos	Weibull	*α* = 0.13214, *β* = 0.58631
	Long videos		*α* = 0.10916, *β* = 0.54129
Backward jump length	Short videos	Weibull	*α* = 0.08108, *β* = 0.59850
	Long videos		*α* = 0.05959, *β* = 0.59236

**Table 2:** Ranking results for 20 candidate schemes.

Ranking	Scheme Num	Anchor Interval	Ranking	Scheme Num	Anchor Interval
1	Scheme 5	2.5 min	11	Scheme 14	7 min
2	Scheme 6	3 min	12	Scheme 15	7.5 min
3	Scheme 7	3.5 min	13	Scheme 16	8 min
4	Scheme 4	2 min	14	Scheme 17	8.5 min
5	Scheme 8	4 min	15	Scheme 18	9 min
6	Scheme 9	4.5 min	16	Scheme 19	9.5 min
7	Scheme 10	5 min	17	Scheme 20	10 min
8	Scheme 11	5.5 min	18	Scheme 3	1.5 min
9	Scheme 12	6 min	19	Scheme 2	1 min
10	Scheme 13	6.5 min	20	Scheme 1	0.5 min

## References

[1] Sheshjavani A.G., Akbari B., Ghaeini H.R. CMPVoD: A cluster mesh-based architecture for VoD streaming over hybrid CDN-P2P networks. Proceedings of the International Symposium on Telecommunications (IST).

[2] Passarella A. (2012). A survey on content-centric technologies for the current Internet: CDN and P2P solutions. Comput. Commun..

[3] Zhong L., Xu C. DLCA: Distributed load balancing and VCR-aware two-tier P2P VoD system. Proceedings of the IEEE International Conference on Consumer Communications and Networking Conference (CCNC).

[4] Liu P., Huang G., Zhou Y., Qin D., Liu S. Server load based prefetching strategy for P2P VoD streaming. Proceedings of the International Conference on Computer Science and Network Technology (ICCSNT).

[5] Riad A.M., Elmogy M., Shehab A.I. (2013). A framework for cloud P2P VoD system based on user’s behavior analysis. Int. J. Comput. Appl..

[6] Xu T., Ye B., Wang Q., Li W., Lu S., Fu X. APEX: A personalization framework to improve quality of experience for DVD-like functions in P2P VoD applications. Proceedings of the 18th International Workshop on Quality of Service (IWQoS).

[7] Aliakbari Z., Fooladi M.D.T. Pre-fetching strategy to support VCR operation in P2P VoD systems. Proceedings of the International Conference on Computer and Knowledge Engineering (ICCKE).

[8] García R., Pañeda X.G., García V., Melendi D., Vilas M. (2007). Statistical characterization of a real video on demand service: User behaviour and streaming-media workload analysis. Simul. Model. Pract. Theory.

[9] Yoon K.P., Hwang C.-L. (1995). Multiple Attribute Decision Making: An Introduction.

[10] Yang G., He S., Shi Z. (2017). Leveraging crowdsourcing for efficient malicious users detection in large-scale social networks. IEEE Internet Things J..

[11] Zhou L., Wu D., Dong Z., Li X. (2017). When Collaboration Hugs Intelligence: Content Delivery over Ultra-Dense Networks. IEEE Commun. Mag..

[12] Liu L., Wang Y.N., Hou L., Feng X.R. (2017). Easy encoding and low bit-error-rate chaos communication system based on reverse-time chaotic oscillator. IET Signal Process..

[13] Yang G., He S., Shi Z., Chen J. (2017). Promoting cooperation by the social incentive mechanism in mobile crowdsensing. IEEE Commun. Mag..

[14] Li X., Wang L., Cui J., Zheng B. A New Fragmentation Strategy for Video of HTTP Live Streaming. Proceedings of the International Conference on Mobile Ad-Hoc and Sensor Networks (MSN).

[15] Guo L., Chen S., Xiao Z., Zhang X. DISC: Dynamic interleaved segment caching for interactive streaming. Proceedings of the 25th IEEE International Conference on Distributed Computing Systems (ICDCS).

[16] Costa C.P., Cunha I.S., Borges A., Ramos C.V., Rocha M.M., Almeida J.M., Ribeiro-Neto B. Analyzing client interactivity in streaming media. Proceedings of the ACM 13th International Conference on World Wide Web.

[17] Wang D., Liu J. (2008). A dynamic skip list-based overlay for on-demand media streaming with VCR interactions. IEEE Trans. Parallel Distrib. Syst..

[18] Ullah I., Doyen G., Bonnet G., Gaiti D. (2012). A survey and synthesis of user behavior measurements in P2P streaming systems. IEEE Commun. Surv. Tutor..

[19] Choi J., Reaz A.S., Mukherjee B. (2012). A survey of user behavior in VoD service and bandwidth-saving multicast streaming schemes. IEEE Commun. Surv. Tutor..

[20] Azibi R., Vanderpooten D. (2002). Construction of rule-based assignment models. Eur. J. Oper. Res..

